# Multi-functional nano silver: A novel disruptive and theranostic agent for pathogenic organisms in real-time

**DOI:** 10.1038/srep34058

**Published:** 2016-09-26

**Authors:** Ponnusamy Manogaran Gopinath, Anandan Ranjani, Dharumadurai Dhanasekaran, Nooruddin Thajuddin, Govindaraju Archunan, Mohammad Abdulkader Akbarsha, Balázs Gulyás, Parasuraman Padmanabhan

**Affiliations:** 1 Department of Microbiology, Bharathidasan University, Tiruchirappalli-620 024, India; 2 National Centre for Alternatives to Animal Experiments (NCAAE), Bharathidasan University, Tiruchirappalli-620 024, India; 3 Centre for Pheromone Technology, Department of Animal Science, School of Life Sciences, Bharathidasan University, Tiruchirappalli-620 024, India; 4 Department of Food Science and Nutrition, College of Food and Agriculture, King Saud University, Riyadh, Kingdom of Saudi Arabia; 5 Lee Kong Chian School of Medicine, Nanyang Technological University, 636921, Singapore

## Abstract

The present study was aimed at evaluating the fluorescence property, sporicidal potency against *Bacillus* and *Clostridium* endospores, and surface disinfecting ability of biogenic nano silver. The nano silver was synthesized using an actinobacterial cell-filtrate. The fluorescence property as well as imaging facilitator potency of this nano silver was verified adopting spectrofluorometer along with fluorescent and confocal laser scanning microscope wherein strong emission and bright green fluorescence, respectively, on the entire spore surface was observed. Subsequently, the endospores of *B. subtilis, B. cereus*, *B. amyloliquefaciens, C. perfringens* and *C. difficile* were treated with physical sporicides, chemical sporicides and nano silver, in which the nano silver brought about pronounced inhibition even at a very low concentration. Finally, the environmental surface-sanitizing potency of nano silver was investigated adopting cage co-contamination assay, wherein vital organs of mice exposed to the nano silver-treated cage did not show any signs of pathological lesions, thus signifying the ability of nano silver to completely disinfect the spore or reduce the count required for infection. Taken these observations together, we have shown the multi-functional biological properties of the nano silver, synthesized using an actinobacterial cell-filtrate, which could be of application in advanced diagnostics, biomedical engineering and therapeutics in the near future.

It was independent discovery of Tyndall, Cohn, and Koch in late 19^th^ century that a few bacterial species spend a part of their life in an inactive (dormant) state called endospore, a renowned hardiest form of life on the earth^1^. These endospores are found in every type of environment, from acid through neutral to alkaline, frozen to hottest, fertile to desert, and fresh and marine water columns to bottom deposits. These spores can keep themselves alive for a remarkably long period. For instance, around 250 million year old *Bacillus* spores from environmental samples^2^, as well as 25 to 40 million year old viable *B. sphaericus* spores from the gut of a fossilized bee in Dominican amber^3^ were retrieved and the organism regenerated. These endospores are extremely resistant to treatments such as harsh chemicals, UV radiation and wet and dry heat that usually kill the vegetative cells^1^,^4^,^5^,^6^. In view of the intrinsic resistance and extended survival, the endospores of *Bacillus* and *Clostridium* species are ideal vehicles for transmission of spore-mediated diseases such as anthrax (*B. anthracis*), gas gangrene (*C. perfringens*), botulism (*C. botulinum*), tetanus (*C. tetani*), food poisoning (*C. perfringens* and *B. cereus*) and pseudomembranous colitis (*C. difficile*) in the health-care domain as well as in bioterrorist and biowarfare attacks^7^,^8^. The currently available chemical disinfectants pose serious limitations in view of their environmental toxicity, corrosivity and insufficient activity at low temperatures. Thus, finding novel sporicidal agents is of utmost concern^9^.

Nanotechnology is a ground-breaking technology that includes pedagogical disciplines viz., material science, physics, chemistry, biology and medicine, and provides newer avenues to prevent and control several diseases by application of nanomaterials. Nanomaterials are gaining importance in cancer therapy^10^,^11^, topical medicine^12^, drug delivery^13^, gene delivery^14^, diagnostic imaging^15^, protein detection^16^, medical devices^17^, tissue engineering^18^, corrosivity alleviation and environmental pollution control^19^. In particular, the noble silver metal has gained enormous attraction due to its unique physical, chemical, mechanical, magnetic, electrical, electronic, thermal, optical and biological properties^20^ and has shown greatly proven inhibitory effects against several microbial pathogens in *in vitro*^21^,^22^,^23^,^24^,^25^,^26^,^27^ as well as *in vivo*^28^ models, mosquito vectors, ticks^29^, etc. Consequently, the nano silver is being commercialized worldwide for its antimicrobial properties in numerous products such as personal care products, medical devices, wound dressings, cosmetics, food preservatives, water filters, washing machines and computer keyboards^17^,^30^,^31^,^32^. However, to-date, a biogenic fluorescent nano silver as well as a nano-based sporicidal agent has not yet been successfully formulated.

Nano silver is generally synthesized by chemical (chemical reduction/pyrolysis), physical (arc-discharge/physical vapor condensation) and biological (plant/microbe-based) methods. In the biological process, nanomaterials are synthesized by assembling atoms/molecules at nanoscale level by means of natural reducing as well as stabilizing agents with less toxicity when compared to those synthesized using chemical and physical methods^20^. Credibly, biogenic nano silver displayed no/less toxic effect on human umbilical vein endothelial cells (HUVEC), Chinese hamster ovary cells (CHO) and a rat cardiomyoblast cell line (H9C2) when compared to chemically synthesized nano silver which exhibits cytotoxic effect even at the minimal dose^11^. Also, a significant inhibition of cancer cell (MCF-7 human breast cancer cell line, B16 mouse melanoma cell line and A549 human lung cancer cell line) proliferation was found as caused by biogenic nano silver compared to the chemical nano silver^11^. Among the various approaches to biological syntheses, actinobacteria are considered the most advantageous and safe compared to other microorganisms as well as plant-based materials, because of the several medicinally important secondary metabolites viz., antibiotics^33^, herbicides^34^, antitumor agents, immunosuppressive agents and other non-toxic extracellular functional molecules that the actinobacteria produce^35^. In this background, the present study was conducted to assess the multi-functional applications such as diagnostic/imaging (fluorescence) property, sporicidal potential and environmental disinfecting ability of actinobacteria-mediated nano silver.

## Results and Discussion

Since antiquity, metallic silver is known for its antimicrobial property and, thus, used as ornaments and utensils by mankind. For example, Egyptians implanted silver plates into the skulls (2500 BC), Greeks and Romans preserved food/liquids in silver containers, and Chinese (659 AD) restored teeth using silver paste^17^ and Indians (7–9 century AD) improved human health using metallic *bhasma*^36^. During the mid-20^th^ century, newly discovered antibiotics superceded large use of silver and silver composites in medicine, food and water. However, the recent emergence and prevalence of antibiotic-resistant microorganisms brought about a revival of metallic silver research. Consequently, today, silver and silver compounds are available for numerous biomedical and therapeutic applications.

**Figure 1:**
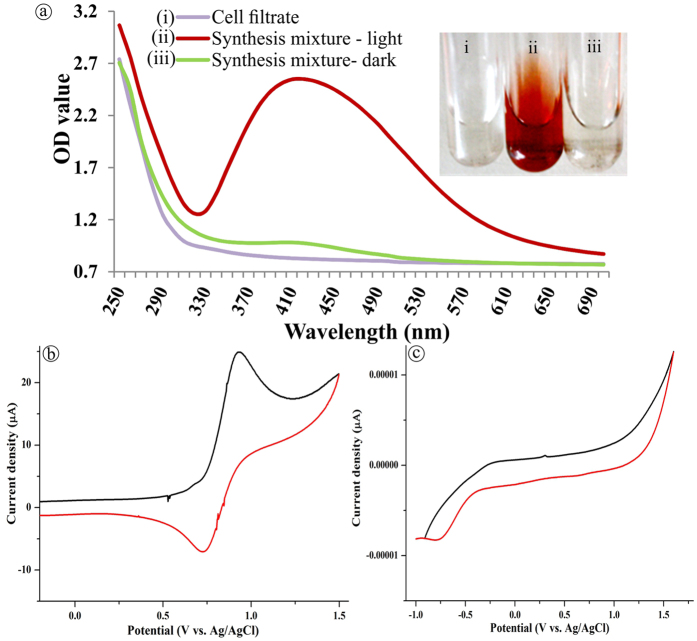
UV- vis spectra and cyclic voltammogram. (**a**) Absorbance spectra of synthesis mixture incubated under light and dark condition. CV of (**b**) aqueous silver nitrate solution (0.5 mM) and (**c**) biogenic nano silver. (Ag/AgCl: Reference electrode; V: Volts).

**Figure 2:**
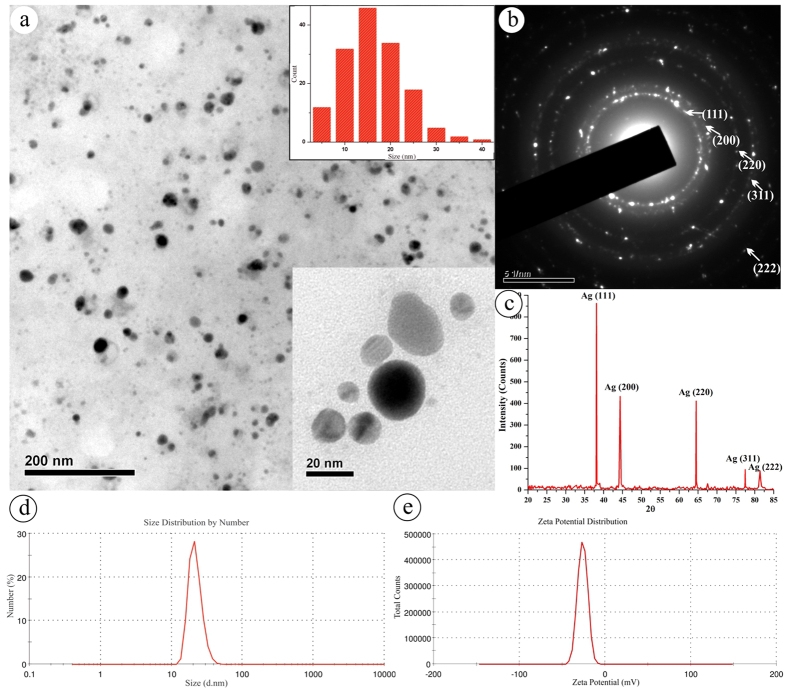
Nano silver characterization. (**a**) HR-TEM micrograph of synthesized nano silver (insert: size distribution histogram of nano silver); (**b**) SAED pattern of nano silver; (**c**) XRD analysis of nano silver; (**d**) Size distribution of nano silver; (**e**) Zeta potential distribution of nano silver.

Sunlight-irradiated rapid synthesis of nano silver using *Streptomyces* sp.- GRD cell-filtrate was successfully performed as described earlier^35^,^37^. Nano silver formation was primarily confirmed by the visible color change of the synthesis mixture from transparent to reddish brown (Fig. 1a, insert ii). This ultimate color change is due to the surface plasmon resonance (SPR) of nano silver which results in a UV-visible spectrum (Fig. 1a) with an absorption maximum at ~410 nm, attributable to the formation of nano sized silver particles. Conversely, neither color change (Fig. 1a, inserts i & iii) nor absorbance peak (Fig. 1a) was observed in the cell-filtrate as well as synthesis mixture incubated at dark. The nano silver formation using actinobacteria^35^, bacteria, fungi^23^ and plant^38^ extracts under the influence of sunlight is very rapid when compared to the dark condition that required hours to days^24^,^39^. The photoreduction mechanism of silver ions (Ag^+^) using biological extracts suggests that sunlight enables decomposition of photosensitive silver nitrate which leads to production of Ag^+^ and at the same time, promotes the interaction of COO^−^ groups present in the synthesis mixture with the Ag^+^, leading to the transfer of electrons^23^,^37^,^40^ which in turn triggers a complete reduction of Ag^+^, and/or the O-H bond undergoes homolytic cleavage to form the hydrogen radical that eventually transfers its electron to the Ag^+^, generating nano silver^38^. Additionally, a complete reduction of Ag^+^ into nano silver and release of Ag^+^ from nano silver was verified using cyclic voltammetry (CV) in which the aqueous silver nitrate solution (Fig. 1b) displayed an oxidation and a reduction peak at +0.72 V and +0.93 V, respectively, validating the electrodeposition of Ag^+^ on the electrode surface and oxidation of silver from the electrode. On the contrary, oxidation and reduction peaks were not observed in the nano silver thus synthesized (Fig. 1c), which could be due to the biomolecular capping on the nano silver surface that would prevent the diffusion of ions from the electrolyte to the electrode surface. This clearly evidences that, during as well as after the nano silver synthesis, the biomolecules of actinobacteria prevent the electron transfer thereby providing the prolonged stability in liquid suspension as well as inhibition of further particle growth by aggregation^37^. High-resolution transmission electron microscopic analysis (HR-TEM) evidences the spherical and slightly elongated nano silver measuring <40 nm (Fig. 2a, insert). Further, selected area electron diffraction (SAED) mode, similar to X-ray diffraction (XRD) analysis but with a higher resolution, was employed to study the crystalline nature of the synthesized nano silver (Fig. 2b), which showed characteristic concentric rings with intermittent bright dots ascribed to (111), (200), (220), (311) and (222) crystalline lattice planes of face-centered cubic nano silver^11^,^41^,^42^ analogous to the sharp XRD Braggs reflection at the 2θ values, 38.12, 44.36, 64.49, 77.45 and 81.35 of silver (Fig. 2c) matching the database of Joint Committee on Powder Diffraction Standards file no. 04–0783^11^,^23^. Further, no characteristic peaks of other crystalline impurities were observed in the entire scanning range which implies the purity of nano silver. The XRD pattern, which has strong (111) Braggs reflection, showed that the sample is rich in Ag nanospheres and, thus, corroborates the outcome of HR-TEM analysis. The XRD spectrum and SAED pattern clearly suggested that the nano silver synthesized using *Streptomyces* sp.- GRD cell-filtrate was crystalline in nature, and concurred with the previous reports^25^,^39^. To determine the particles size distribution in solution, quasi-elastic light scattering or dynamic light scattering (DLS), that quantifies the particle’s nucleus size, surface structures and concentration, was employed. The size distribution of the synthesized nano silver ranged from 15 to 52 nm (Fig. 2d), with polydispersity index (PDI) of 0.356 signifying the moderately polydispersive nature^43^. This size distribution profile of DLS had close correlation with HR-TEM (histogram). Stability of nano metals is an important requisite for their several biomedical and other applications. However, nano metals generally tend to aggregate over time owing to the high surface energy^44^. Therefore, zeta potential measurement was carried out for a period of 6 months to assess the stability of the nano silver in the required medium. As reported by Rajput and coworkers the proteinaceous-corona layer around the nano silver leads to a negative zeta potential (−30.2 ± 0.8 mV) (Fig. 2e) and affords the stability by either electrostatic repulsion or steric effects or a combination of both. The nano material with the surface charge of ≤–30 mV and ≥+ 30 mV is stable from aggregation and precipitation^45^. Obviously, this sustained stability of the nano silver could be due to the complete reduction of Ag^+^ as well as the effective biomolecular capping by the actinobacterial metabolites^11^,^37^,^46^.

**Figure 3:**
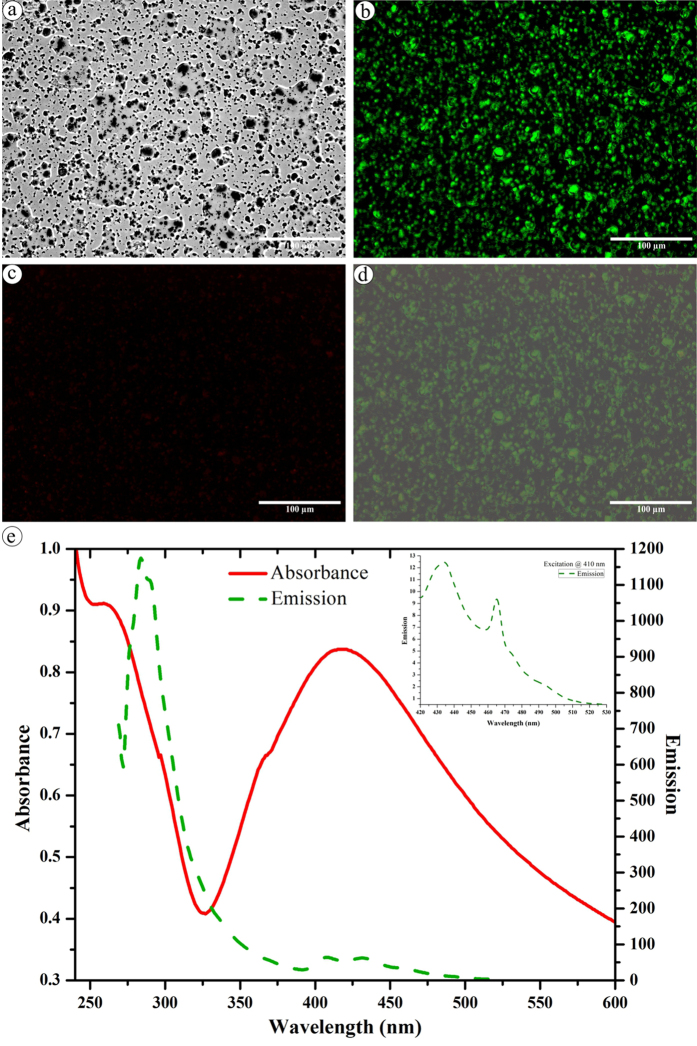
Fluorescence behavior of synthesized nano silver. (**a**) Image under transmitted light; (**b**) Green fluorescent image; (**c**) Red fluorescent image; (**d**) a, b and c superimposed; (**e**) Absorption and emission spectra of nano silver (insert: emission spectra at 410 nm excitation).

Fluorescent nano materials are remarkable imaging probes since their detection is not limited by the Rayleigh scattering condition. An absolute light emission arises either from the molecule’s surface or small Ag clusters but not from large silver particles^47^,^48^. However, fluorescence and Raman scattering are strong in larger silver clusters^49^ and this signal-enhancement may be influenced by the nano silver’s distance, relative orientation of emitter with local electric field shaped by the nano silver^49^,^50^, and the chemical bond with the nano silver^51^,^52^. Surprisingly, we detected that the drop-coated dried nano silver solution emitted strong green and weak red fluorescence spots when excited with continuous-wave of green and red fluorescent LED light, respectively (Fig. 3b,c). Approximately 25 μm thick rim of materials was observed at the edge of the droplet whereas in the center part silver aggregates (due to drying process) were fairly sparse and almost all the observed visible structures exhibited a fractal-like shape. Further, the absorbance spectrum of nano silver solution displayed peaks at ~410 and 259 nm (Fig. 3e), corresponding to the SPR of nano silver and the biological capping molecules, respectively. Appearance of peak at 259 nm could be due to removal of biomolecules other than the capping agent that hinders its absorption of capping molecules from the synthesis mixture. When exited at 259 nm a strong emission was observed at 289 nm (Fig. 3e) and when excited at 410 nm a primary as well as secondary emission peaks were observed at 434 nm and 465 nm, respectively (Fig. 3e, insert). The higher emission at 289 nm than at 434 nm indicates that the fluorescence is greatly influenced by the biomolecules present on the nano silver surface. In fact, the unexpected bright fluorescence from the biogenic nano silver attracts attention since it could possibly be used in imaging and diagnostic applications.

**Figure 4:**
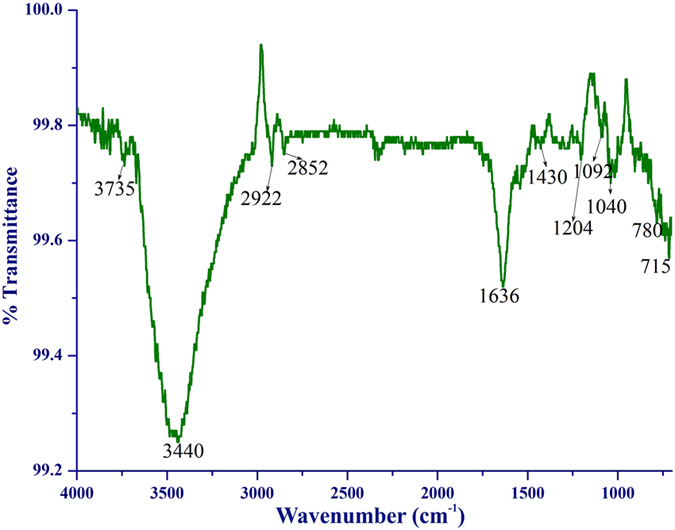
FT-IR analysis of fluorescent nano silver.

In order to find the major biomolecules responsible for the synthesis and stability of the particles and possible fluorescing molecules that are present on the surface of nano silver, Fourier Transform Infrared (FT-IR) spectroscopy was performed for the dried nano silver (60 °C). The spectrum (Fig. 4) showed distinct peaks at 3735, 3440, 2922, 2852, 1636, 1430, 1204, 1092, 1040, 780, and 715 cm^−1^. The absorption band at 3735 cm^−1^ may arise due to the O-H stretching of protein molecules^53^. The strong band at 3440 cm^−1^ corresponds to the free symmetric and asymmetric N-H stretching vibration of 1° (−NH_2_) and 2° amine (−NH-) bonds of proteins^54^. The bands at 2922 cm^−1^ and 2852 cm^−1^ could be attributed to the antisymmetric and symmetric stretching of CH_2_ of lipids as well as some contribution from proteins^55^,^56^. The medium absorption band at 1636 cm^−1^ corroborates the formation of −NH_3_^+^ groups due to the complexation of amino groups and carboxylic groups^23^,^57^. The absorption bands at 1092 cm^−1^, 1040 cm^−1^ and 1204 cm^−1^ corresponds to the O-H and C–O stretching vibration of carboxylic groups^23^,^58^. In addition, there were weak peaks at 1430 cm^−1^ and, 780 cm^−1^ and 715 cm^−1^ which corresponds to the C-H and N-H symmetric deformation of amide II^59^ and C-H bending of aromatic ring, respectively. In association with the FT-IR spectrum of the synthesized nano silver, major functional groups such as carbonyl, hydroxyl and amino group of protein followed by the minor lipid bonds were observed on the surface of the nano silver. The FT-IR peaks of protein and lipid molecules and the absorbance band near 260 nm, attributable to aromatic residues as well as disulfide bonds of proteins, suggest that these molecules may be mainly responsible for the nano silver formation, prevention of aggregation and the fluorescence property. Several FT-IR reports^25^,^37^,^40^,^45^ have demonstrated the involvement of carbonyl groups in the amino acid residues and peptides of proteins for the nano silver synthesis. These protein molecules form a protein coat on the nano silver surface which prevents the agglomeration and provides stability to the nano silver in the aqueous medium^11^.

**Figure 5:**
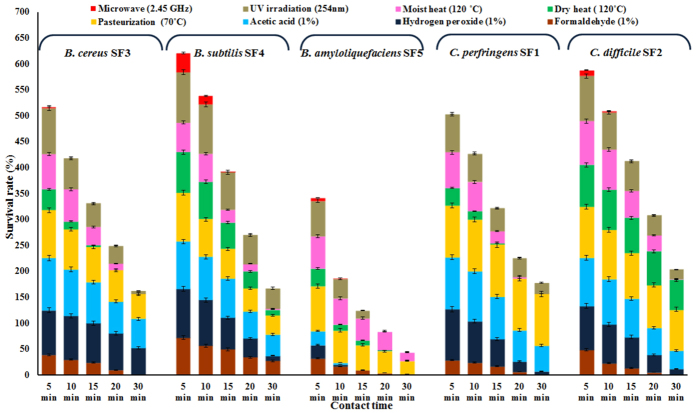
Clustered stacked bar chart for spore survival against selected physical and chemical sporicides.

The endospores of *B. subtilis, B. cereus*, *B. amyloliquefaciens, C. perfringens* and *C. difficile* were isolated from hospital environment and tested for their survival at different time intervals against physical (moist heat, dry heat, pasteurization and UV irradiation) and chemical (hydrogen peroxide, formaldehyde and acetic acid) sporicidal agents. The significant variation in the survival pattern of *Bacillus* and *Clostridium* spores at 5, 10, 15, 20 and 30 min of treatment with selected sporicides are shown in Fig. 5, and the variation could be due to well-organized as well as diverse resistance mechanisms of endospores towards the respective treatments. The factors of resistance include, (i) core water for moist heat and peroxides resistance, (ii) α/β- SASP for UV radiation, dry heat, alkylating agents, formaldehyde and peroxides resistance, (iii) relative impermeability of spore coat for resistance to chemicals, and (iv) genetic makeup and spore repair mechanisms are peculiar for spore resistance^1^,^60^.

Preference Ranking Organization METHod for Enrichment of Evaluations (PROMETHEE) is a non-parametric multivariate ranking procedure in which all objects and variables are analyzed simultaneously as well as systematically thus validating a matrix containing small number of samples. Previously, multi-criteria decision aid (MCDA) technique has been employed for ranking antifungal property of organotin (IV) compounds^61^ and biodiesel production from cyanobacteria^62^. In this work, we intended to rank the spore survival against the tested chemical and physical sporicides using endospores as actions and the sporicides such as formaldehyde (1%), H_2_O_2_ (1%), acetic acid (1%), pasteurization (70 °C), dry heat (120 °C), moist heat (120 °C), UV irradiation (254 nm) and microwave (2.45 GHz) as variables. Therefore, the percentages of spore survival after 10 min treatment were fed to the visual PROMETHEE 1.4 Academic Edition software [developed by Dr. Bertrand Mareschal (2011–2015)] for MCDA analysis with the ‘maximized’ preference, because higher the survival value higher the resistance (Table S1).

**Figure 6:**
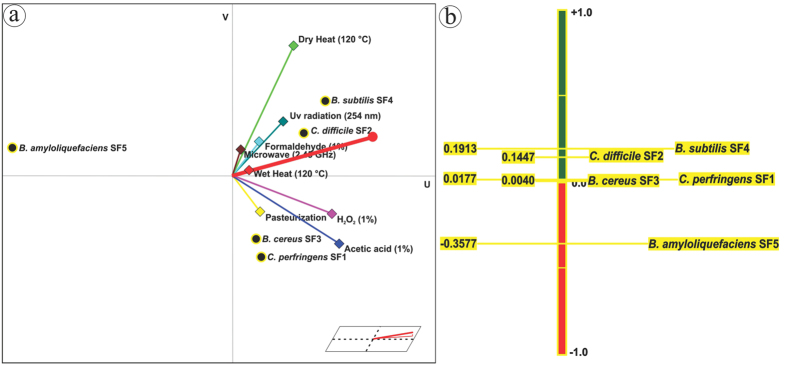
GAIA biplot and PROMETHEE II complete ranking. (**a**) GAIA biplot of *Bacillus* and *Clostridium* spores survival rate generated against selected sporicides; (**b**) Complete ranking of spores based on their outranking flow.

The graphical analysis for interactive aid (GAIA) is the principal component analysis biplot that exhibited approximately 91.5% of the variance gathered by first (U) and second (V) principal components (Fig. 6a). The decision vector (red line), which is influenced mainly by the direction and length of criteria vectors, specified the most preferable action^62^. In general, the actions that are aligned in the direction of decision vector and the outermost criteria in that direction are the most preferable factors^63^. Accordingly, the rate of survival against microwave, formaldehyde, H_2_O_2_ and UV irradiation was higher in *B. subtilis* spores; heat survival was greater in *C. difficile* spores; acetic acid and pasteurization resistance was higher in *C. perfringens* spores, but *B. amyloliquefaciens* spores showed the least survival towards all the cidal agents. Further, the PROMETHEE II complete ranking based on the preference (Phi) net flow which is the balance (difference) between Phi^+^ and Phi^−^ are shown in Fig. 6b in which the preferred highest to least survival of spores against the tested sporicides are *B. subtilis* (ϕ^+^:0.191) >*C. difficile* (ϕ^+^:0.145) >*C. perfringens* (ϕ^+^:0.018) >*B. cereus* (ϕ^+^:0.004) >*B. amyloliquefaciens* (ϕ^−^:0.358). This clearly validates that *B. subtilis* and *C. difficile* spores are more resistant than *C. perfringens, B. cereus* and *B. amyloliquefaciens* spores against the tested physical and chemical cidal agents.

**Figure 7:**
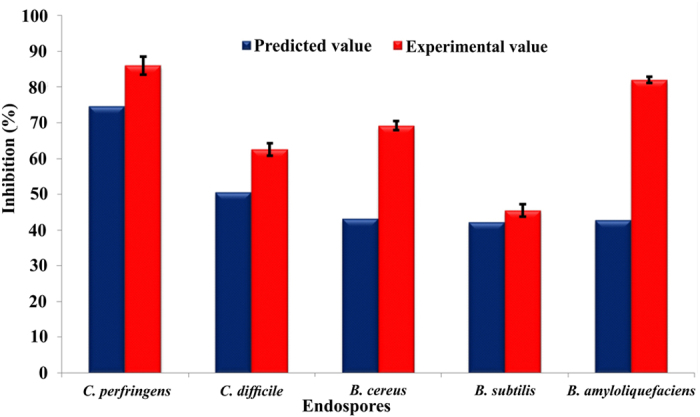
Experimental (10 min) and predicted (8 min) spore inhibition percentage of nano silver under optimized conditions.

**Figure 8:**
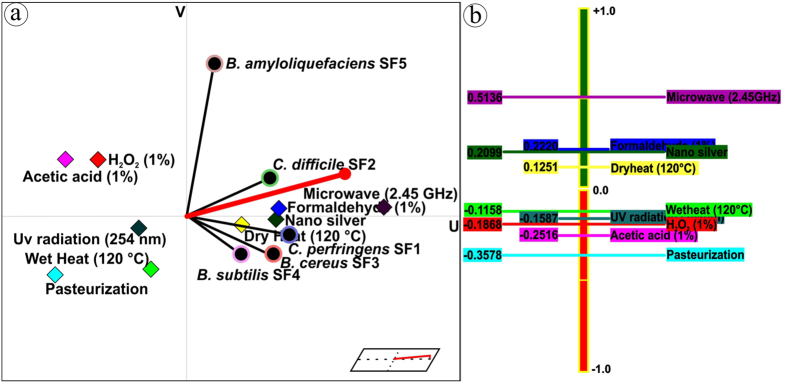
GAIA biplot and PROMETHEE II complete ranking. (**a**) GAIA biplot for sporicidal activity of selected sporicides including nano silver; (**b**) Corresponding ranking of sporicides based on their outranking flow.

In order to accelerate the sporicidal activity, all the influential process parameters (pH, temperature, nano silver concentration and treatment time) were optimized systematically using response surface methodology (RSM), a collection of mathematical and statistical techniques (Tables S2 and S3; Fig. S2) (detailed optimization procedure is presented in the Supplementary Material). As soon as the optimized sporicidal conditions such as temperature (35 °C), pH (6) and nano silver (75μg mL^−1^) (Table S4) were obtained from central composite design under RSM, the average sporicidal efficacy of nano silver at 10 min (from three independent experiments) was estimated and presented along with the predicted inhibition (response value predicted by the model for the experimental conditions) in Fig. 7. Once again, MCDA was performed to rank the sporicidal efficacy of nano silver along with the tested physical and chemical sporicides at 10 min exposure by considering endospores as variables and the nano silver, physical sporicides and chemical sporicides as actions (Table S5). The resulting GAIA biplot exhibited approximately 89.1% of the variance described by the first two principal components (Fig. 8a). The successive PROMETHEE II complete ranking (Fig. 8b) is microwave 2.45 GHz (ϕ^+^:0.514) >formaldehyde 1% (ϕ^+^:0.222) ≥nano silver (ϕ^+^:0.210) >dry heat 120 °C (ϕ^+^:0.125) >moist heat 120 °C (ϕ^−^:0.116) >UV irradiation 254 nm (ϕ^−^:0.159) >H_2_O_2_ 1% (ϕ:0.187) >acetic acid 1% (ϕ^−^:0.252) >pasteurization 70 °C (ϕ^−^:0.358). Among the sporicides tested, microwave was the most preferable cidal agent followed by formaldehyde and nano silver at almost similar preference levels. Though microwave and formaldehyde ranked just ahead of nano silver, these techniques are not preferred so much as nano silver in view of their inappropriateness in biomedical fields. Therefore, we suggest that nano silver could possibly be a new approach to destroy spores in the health care scenario and food processing industry.

**Figure 9:**
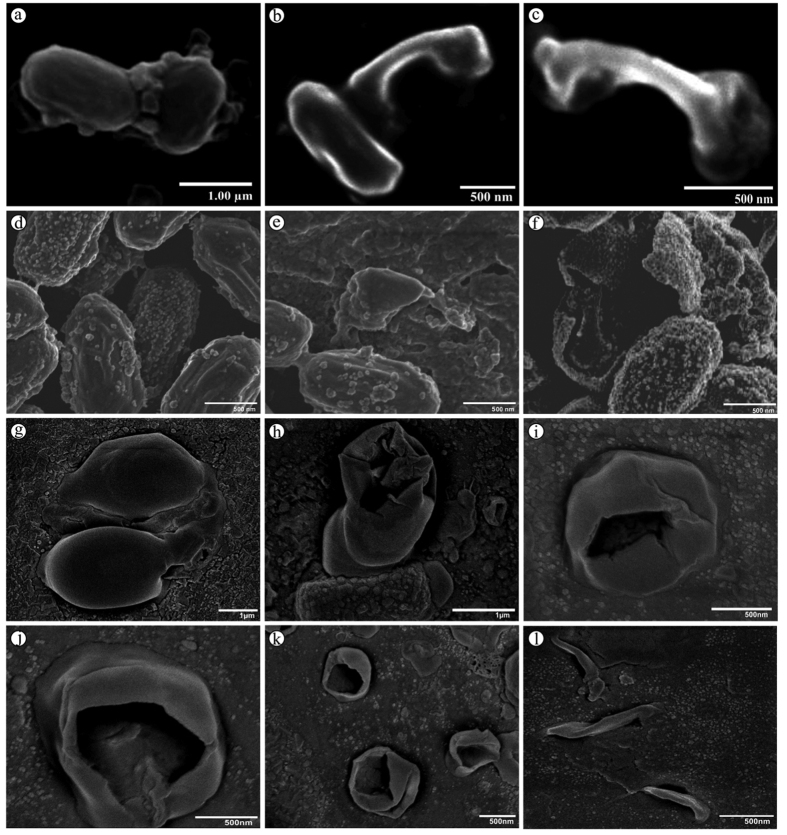
Microscopic examination of nano silver-treated spores. Scanning electron micrograph of *B. subtilis* (**a–c**), HR-FE-SEM micrograph of *B. cereus* (**d–f**) and *C. difficile* (**g–l**) spores exposed to nano silver.

High resolution cold field-emission scanning electron microscopic (HR-FE-SEM) images illustrate the structural deformations in *B. subtilis* (Fig. 9a–c), *B. cereus* (Fig. 9d–f) and *C. difficile* (Fig. 9g–l) spores treated with nano silver. As described above, nano metals, especially silver, have affinity towards proteins, lipids, carbohydrates and other biomolecules. The interaction occurs mainly at (i) either/both the N-terminus (amino nitrogen-donor) and C-terminus (oxygen atoms) in amino acids and proteins, (ii) thiol groups (-SH) and disulfide bonds (R-S-S-R) in enzymes, and (iii) soft acid-base reaction with the sulfur and phosphorus of biomolecules^17^,^23^,^64^. Interestingly, depending on the spore-formers, the outer-most layer of the spore viz., exosporium and/or spore coat are mainly made up of proteins which constitute around 50% of the dry weight, followed by lipids, carbohydrates and phosphorus^4^. These proteins are especially rich in sulphur-containing amino acids, cysteine and methionine, followed by other least amino acids such as histidine and tyrosine^65^. Hence, the adherence of nano silver on the entire spore coat (Fig. 9a,d–f) followed by cut and pit formation (Fig. 9b,e,h–k) due to denaturation of protein as well as the β−1 → 4 glycosidic bonds of the peptidoglycan N-acetylglucosamine and N-acetylmuramic acid^66^ and finally complete structure loss (Fig. 9c,f,l) were recorded. Similar deformation by nano materials was observed on the vegetative *E. coli*^11^ and *S. aureus* cells^67^.

**Figure 10:**
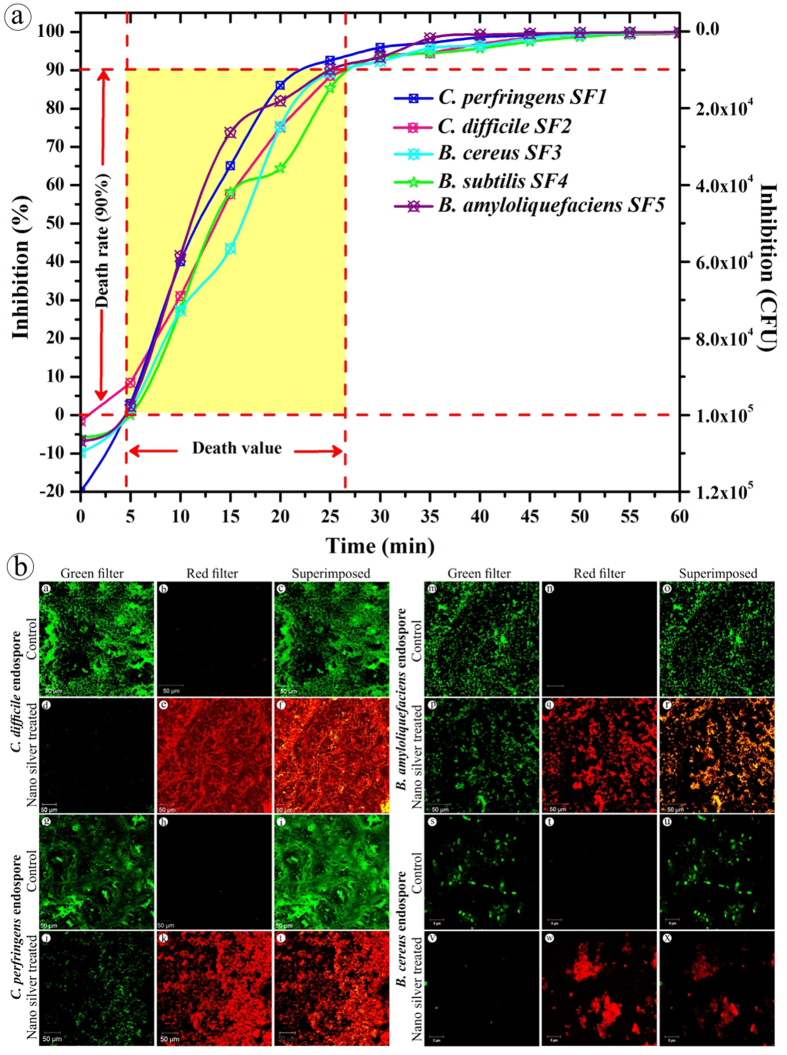
Death value determination of nano silver at RSM optimized condition. (**a**) Inhibition curve of nano silver-treated spores; (**b**) Pre- and post- nano silver-treated *Bacillus* and *Clostridium* spores- CLSM images that are identical.

Ahead of the environmental spore inactivation experiment, death value i.e., the total time required to kill about 90% of viable cells, needs to be determined. Under optimized condition, the 90% spore inhibition was assessed by plotting the percentage of spore survival against time (Fig. 10a). The average D values obtained for *B. cereus*, *B. amyloliquefaciens*, *B. subtilis, C. difficile* and *C. perfringens* spores treated with nano silver were around 20 min. Certainly, spores are hardier than their vegetative form and, thus, their inhibition requires high concentration and increased contact time. For example, the 90% inhibition of *Bacillus* and *Clostridium* endospores was achieved by treatment with chemical disinfectants such as glutaraldehyde (20 mg/mL), sodium hypochlorite (0.25 mg/mL), H_2_O_2_ (15 mg/mL) and formaldehyde (5 mg/mL) in 25, 20.6, 55.2 and 11.8 min, respectively^68^. However, the biogenic nano silver (75 μg/mL) showed more than 90% inhibition at 20 min of treatment. This inhibitory effect of nano silver at comparatively lower concentration than the chemical sporicides could be useful for disinfecting the hazardous spores during biowarfare or bioterrorist attack.

Furthermore, confocal laser scanning electron microscope (CLSM) was employed to evaluate the complete disinfection of spores by nano silver. The pre- and post- nano silver-treated *Bacillus* and *Clostridium* endospores were stained by acridine orange (AO) and ethidium bromide (EB) method (AO- 3 μg/mL & EB- 10 μg/mL) for ~5 min, excess stain was washed with sterile deionized distilled H_2_O and the spore pellet was coated on a fresh glass slide prior to examination. Generally, AO stains nuclei to fluoresce green through its intrinsic permeability to all cells, besides showing high affinity towards acidic polysaccharide-protein matrices^69^. Certainly, endospores possess a similar type of matrix in their thick peptidoglycan cortex below the outer proteinaceous spore coat and, hence, only the cortex region is poorly stained^70^. However, EB is not-permeable into cells until their protective membranes are damaged to stain the nucleus red. CLSM identical images (Fig. 10b) of pre- (a–c, g–i, m–o, s–u) and post- (d–f, j–l, p–r, v–x) nano silver-treated (20 min) *Bacillus* and *Clostridium* spores were generated by FITC band pass filter (which visualizes only the live spores as green fluorescence) and Alexa 594 band pass filter (detects the dead spores as red fluorescence), respectively. The superimposed images distinguished live and dead spores from injured/dying spores qualitatively. More than 90% of spores were inhibited at 20 min treatment with nano silver which was visually confirmed by randomly observing five different fields from each slide.

**Figure 11:**
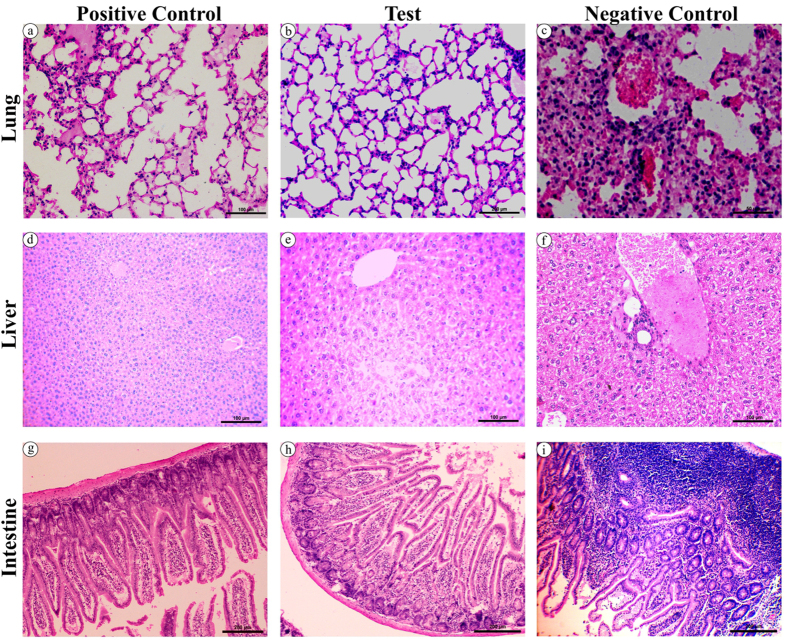
Histopathological evaluation of vital organs of mice exposed to nano silver-treated and -untreated spore co-contaminated cages. H&E-stained sections of lung (**a–c**), liver (**d–f**) and intestine (**g–i**). Positive control – uninfected mice, test – mice exposed to the spores treated with nano silver, and negative control- mice exposed to nano silver -untreated spores.

It is customary that any disinfectant is proved of its ability to sterilize environmental spores and, therefore, the disinfecting potency of nano silver was evaluated by environmental spore co-contamination technique (schematic representation, Fig. S3). For this experiment, the cages were contaminated with 1 × 10^6^ spores of *B. cereus* and *C. difficile,* which are opportunistic pathogens causing cellulitis, bacteremia, meningitis and infectious diarrhea to newborn and immunocompromized patients, and their pathogenicity is manifested mainly by gastrointestinal (GI) or non-gastrointestinal tissue destruction by means of numerous enterotoxins as well as exoenzymes. Moreover, *B. cereus* is a close relative of *B. anthracis*, and produces inhalation anthrax (wool sorters disease)-like infections^71^ as well as lung, liver and spleen infections in mice^72^. In the present study, mouse model was used to evaluate nano silver spore disinfecting ability based on the magnitude of infection. On the 10^th^ day post-exposure to nano silver -treated and -untreated co-contaminated cages, mice were euthanized, the fur was shaved off and carefully examined for inflammation of skin, lesions, abscesses, lumps and scratch- or bite wounds. None of the symptom was observed on the mouse skin and paw; however, diarrheal syndrome was observed in the negative control mice (infected) and, therefore, the liver, intestine and lung (to assess lung infection) were dissected out. Subsequently, the architectural and pathological changes in the lung, liver and gastrointestinal tract of mice exposed to co-contaminated cage (negative control) and nano silver sterilized co-contaminated cage (test) were inspected by a skilled pathologist. The cage without spore contamination was used as positive control. The positive control (Fig. 11a,d,g) and test (Fig. 11b,e,h) mice revealed no pathological changes, while the mice infected with spores of *B. cereus* and *C. difficile* exhibited intra-alveolar, intra-bronchiolar, intra-septal and interstitial accumulation of polymorphonuclear cells and macrophages in the infected lung (Fig. 11c). Moreover, bacteria-like structures within lung interstitium and capillaries were also observed as focally distributed (Fig. S4)^72^, endorsing the development of peribronchial pneumonia and bronchopneumonia in mice which concurs with the pathological findings produced by the intranasal administration of *B. cereus*^72^, *B. anthracis* and *B. subtilis*^73^. However, no such structures were found in the test- and positive-control mice. Likewise, the liver sections of negative control mice showed mixed lympho-monocytic infiltrations around the portal vein and deformation of hepatic parenchyma (Fig. 11f). Furthermore, the GI tract of the negative control mice showed signs of inflammatory cell infiltration, submucosal edema, mucosal damage, ulcerations, hyperplasia, crypt loss and fibrosis (Fig. 11i) similar to the report with regard to *C. difficile*^74^ and *B. cereus*^75^ infections. Throughout the experiment period inflammatory exudate, diarrheal symptoms and gradual increase in the spore count were found in the feces shedding of negative control mice. The absence of pathological lesions in nano silver-treated co-contaminated cage mice could be due to thorough disinfection or reduction in the spores that is vital in causing disease. These pathological results established the first pitch towards application of nano silver as a surface disinfectant against environmental spores.

In general, disinfectants that are commonly used to sterilize equipment and floors of hospital, dairy and food packaging industries destroy pathogens either by attacking them from outside or from within, wherein the exterior disinfectants cause cell disruption^76^,^77^. Based on the APIC guidelines there are several disinfectants which, even at very high concentrations, fail to destroy the bacterial endospores^78^. Since the present study has shown that nano silver disrupts the spores as well as arrests the ability of spores for revival, similar to the exterior decontaminating agents, it could be potentially used as a disinfectant during the spore outbreaks in bioterrorism or biowarfare attack, apart from disinfection of spores and pathogenic microorganisms in equipment and health care environment as well as food processing industries.

**Figure 12:**
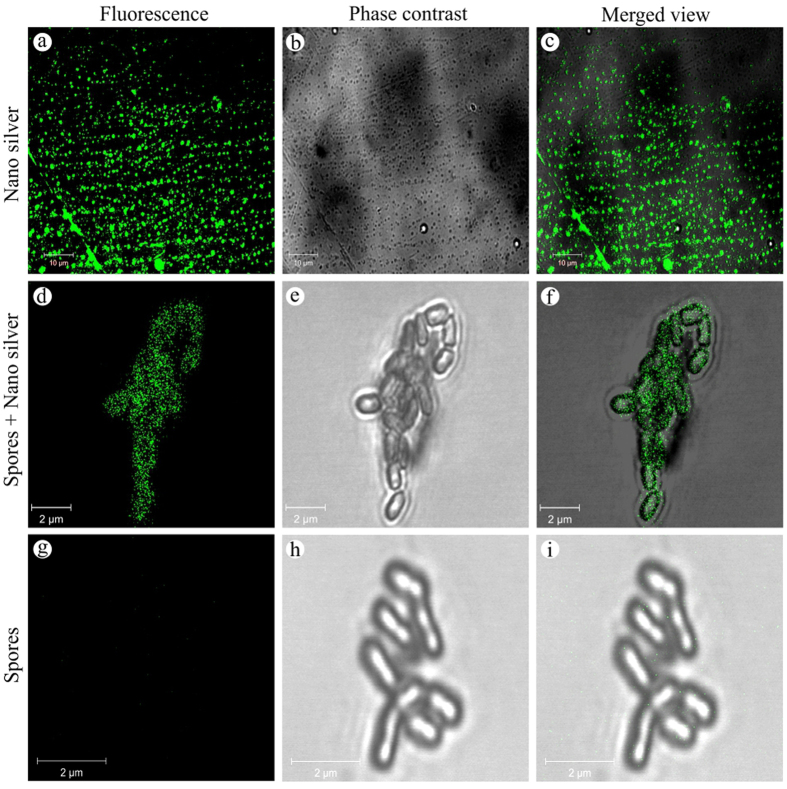
Fluorescence and the corresponding phase contrast images of nano silver and nano silver pre- and post-treated spores observed in CLSM.

**Figure 13:**
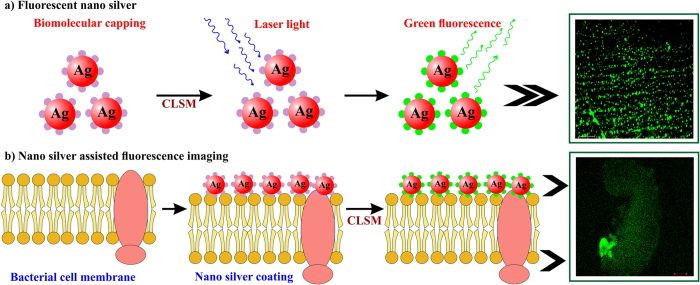
Graphical illustration of nano silver coating on spore surface and fluorescence of entire spore structure.

Recently, the fluorescence property of biogenic nano silver opened up a newer avenue in diagnostic and imaging applications. The guaranteed fluorescent property of biogenic nano silver inspired us to examine nano silver-treated spores in a CLSM^79^, wherein a characteristic strong green fluorescence emanated from nano silver itself (Fig. 12a) and from the entire surface of spores treated with nano silver (Fig. 12d) when excited at 458 nm. This fluorescence of entire spore structure could be due to the thorough coating by nano silver as illustrated in Fig. 9d,f, and Fig. 13. In contrast, no fluorescence was found on control spores (Fig. 12g). Previously, the fluorescent nano silver-based diagnosis and imaging were described by adopting CLSM^79^ and fluorescent microcopy^11^. This unexpected strong green fluorescence in fluorescent microscope as well as CLSM evidences that biogenic nano silver could possibly be applied in various biomedical imaging and diagnostics applications in the near future.

## Methods

### Nano silver synthesis and characterization

The *Streptomyces* sp*.-* GRD [ACCN: JX512257] cell-filtrate was challenged with silver nitrate to a final concentration of 0.5 mM under direct sunlight. The synthesized nano silver was characterized using UV-vis spectrophotometer (SHIMADZU-UV-1800, Japan), HR-TEM (JEOL JEM 2100, Peabody, MA, USA) and SAED pattern analysis. The XRD pattern was documented with the X’Pert Pro, PANalytical, Westborough, MA, by operating at 40 kV with a current of 30 mA and Cu Kα radiation (l = 1.54 Å) from 25 to 85° 2θ angles. Subsequently, the synthesized nano silver was separated by centrifugation and investigated under a PC controlled electrochemical analyzer containing glassy carbon (working electrode), Pt wire (counter electrode) and Ag/AgCl (reference electrode) for the complete reduction/release of Ag^+^. Dried nano silver was ultrasonicated in deionized distilled water before dynamic light scattering and particle size distribution profile studies under Zetasizer Ver. 6.20 (MAL1052893), Malvern Instruments Ltd. Further, the fluorescence behavior was inspected in a fluorescent microscope (EVOS™ FLoid™ Cell Imaging Station, Life Technologies, Carlsbad, CA) and CLSM (Zeiss LSM 710, Carl Zeiss, Germany), for which the heat-dried nano silver was dissolved in sterile deionized distilled water and drop coated on a clean glass slide at 60 °C. Subsequently, spectrofluorometer (JASCO FP-8600) was employed for fluorescence measurements in which the nano silver solution was excited at 259 and 410 nm, and the emission spectra was observed in the range 250–600 nm.

### Isolation of spores and endospore preparation

Endospores of *B. subtilis* [ACCN: JX850070], *B. cereus* [ACCN: KM403375], *B. amyloliquefaciens* [ACCN: KM403376], *C. perfringens* [ACCN: KU236372] and *C. difficile* [ACCN: KU236371] were isolated from the preheated (65 °C) soil and sewage samples collected from the hospital environment of Tiruchirappalli, Tamil Nadu, India. Retrieved spores were subsequently cultured in brain-heart infusion (BHI) agar, and spore formation was documented (Fig. S1). Endospores were prepared in reinforced Clostridial broth (*Clostridium* sp.) and sporulation medium (*Bacillus* sp.) by incubating 8 weeks in dark^37^.

### MCDA analysis of spore survival to physical and chemical sporicidal agents

The rate of spore survival in chemicals [formaldehyde (1%), hydrogen peroxide (1%) and acetic acid (1%)], temperatures [pasteurization (70 °C), dry heat (120 °C) and moist heat (120 °C)], and radiation [UV irradiation (254 nm) and microwave (2.45 GHz)] was evaluated by treating spores for 5, 10, 15, 20 and 30 min time interval with the respective sporicide. The percent spore survival after 10 min were fed to Visual PROMETHEE software v1.4.0 for MCDA analysis using PROMETHEE and GAIA algorithms to rank the spores according to survivability/tolerance.

### Assessment of nano silver sporicidal activity

The parameters namely, pH, temperature, nano silver concentration and treatment time were controlled by adopting RSM for enhanced sporicidal potency against spores (Tables S2 and S3 and Fig. S2). Under optimized condition obtained from central composite design (CCD) design of RSM (Table S4), sporicidal potency of nano silver was tested and the percentage of killing after 10 min contact time was estimated.

### Microscopic examination of sporicidal activity

Morphological impairment of *B. subtilis, B. cereus* and *C. difficile* spores treated with nano silver were examined in cold HR-FE-SEM (JEOL, JSM-6701F) at 3.0 kV by fixing a thin film of spore-nano silver mixture on copper grid and subsequently coating with gold in a sputter coater.

### Environmental spore decontamination studies of nano silver

#### Determination of decimal reduction time

Under RSM-optimized condition, the death value of the endospores treated with nano silver was estimated by transferring the spore suspension (1.0 mL) in 99 mL of nano silver solution (final conc. 10^5^ CFUmL^−1^). One milliliter of the aliquot was transferred and serially diluted in 9 mL of sterile BHI broth at regular time intervals (5 min). Reduction in spore count was determined by plating 100 μL aliquot from respective dilution onto BHI plate. This procedure was triplicated for all the endospores separately and the average reduction in colony-forming unit (CFU) from the triplicates was used for the determination of death values for *Bacillus* and *Clostridium* endospores.

#### Determination of live and dead spores

CLSM (Zeiss LSM 710, Carl Zeiss, Germany) was employed to observe live and dead spores. Briefly, AO and EB were used to stain the endospores treated with and without nano silver for ~5 min^80^. Green and red fluorescence of AO and EB, respectively, were observed under band pass filter ranging between 505 and 530 nm, and 585 nm long pass filter, respectively, with 488 nm excitation^37^.

#### Determination of environmental spore disinfection

The ability of nano silver to decontaminate environmental spores was validated by adopting cage-co-contamination technique (Fig. S3). Under sterile condition, a mixture of (10^6^ CFU) *B. cereus* and *C. difficile* spores were introduced onto sterile cages (~1000 spores/cm^2^) 18 hr preceding the experiment. At the time of experiment, about 15 mL of nano silver solution was added to co-contaminated cage (test cage) and allowed to decontaminate the spores for 20 min. Later, the sporicidal suspension was removed by patting the surface with sterile paper towels. Similarly, the solution without nano silver was added to another co-contaminated cage (negative control). Subsequently, mice were aseptically placed in test and negative control cages for an hour and aseptically transferred to sterile separate cages. Besides, cage without spore served as a positive control. The animal infection experiment was approved by the Institutional Animal Ethics Committee, Bharathidasan University (BDU/IAEC/29/2013/09-04-2013) and executed according to the guidelines of Committee for the Purpose of Control and Supervision on Experiments on Animals (CPCSEA).

### Histopathological evaluation

After incubation, mice were anesthetized using sodium pentobarbital, sacrificed by cervical dislocation and organs were removed immediately according to the CPCSEA SOP for laboratory experimental animals. Lung, liver and intestine were quickly dissected out, washed with PBS (pH 7), fixed in 10% neutral buffered formalin and processed for paraffin wax embedding. Five micron thick sections were stained in hematoxylin and eosin using Rapid H&E staining kit (BioLab Diagnostics, Tarapur, India) according to manufacturer’s instructions to observe the histopathological changes, if any, in a Nikon Eclipse TS100 Microscope (USA).

## Conclusion

To conclude, our study reports a novel fluorescent nano silver synthesized using *Streptomyces* sp.-GRD, and its imaging facilitator potency as well as ability to destroy bacterial endospores. *Bacillus* and *Clostridium* spores were isolated from health-care environment, treated with physical and chemical sporicides and their survival rate was ranked. Subsequently, biologically derived nano silver was tested against endospores under optimized condition, wherein a complete structural loss of spores was documented. The environmental spore disinfectant potency of nano silver was demonstrated using mouse model, in which no sign of pathological lesions was observed after nano silver sterilization. Nano silver-based fluorescent imaging of endospores was verified under CLSM. Based on these findings, we recommend that the *Streptomyces* sp.-GRD-mediated nano silver could possibly be applied as a surface disinfectant against environmental spores as well as for several theranostic applications. However, further investigations with regard to *Streptomyces* sp.-GRD-mediated nano silver synthesis, fluorescence biomolecular capping agent, as well as environmental decontamination technique are required prior to these and other applications.

## Additional Information

**How to cite this article**: Gopinath, P. M. *et al.* Multi-functional nano silver: A novel disruptive and theranostic agent for pathogenic organisms in real-time. *Sci. Rep.* **6**, 34058; doi: 10.1038/srep34058 (2016).

## Supplementary Material

Supplementary Information
