# Supplementary material for: Multi-functional nano silver: A novel disruptive and theranostic agent for pathogenic organisms in real-time
Source: Sci Rep. 2016 Sep 26;6:34058. doi: 10.1038/srep34058 (PMC5036090; doi:10.1038/srep34058)
Supplement: Supplementary Information [file srep34058-s1.doc]

**Supplementary materials**

**Multi-functional nano silver: A novel disruptive and theranostic agent for pathogenic organisms in real-time**

P. M. Gopinath, A. Ranjani, D. Dhanasekaran, N. Thajuddin, G. Archunan, M. A. Akbarsha, Balázs Gulyás and P. Padmanabhan

**Figure S1. Schaeffer- Fulton endospore staining images (**a) *C. perfringens*, (b) *C. difficile*, (c) *B. subtilis*, (d) *B. cereus*, (e) *B. amyloliquefaciens* and (f) pure spores (*B. amyloliquefaciens*) under Micros microscope (Austria) equipped with a digital camera. All the images were taken at 100x magnification.


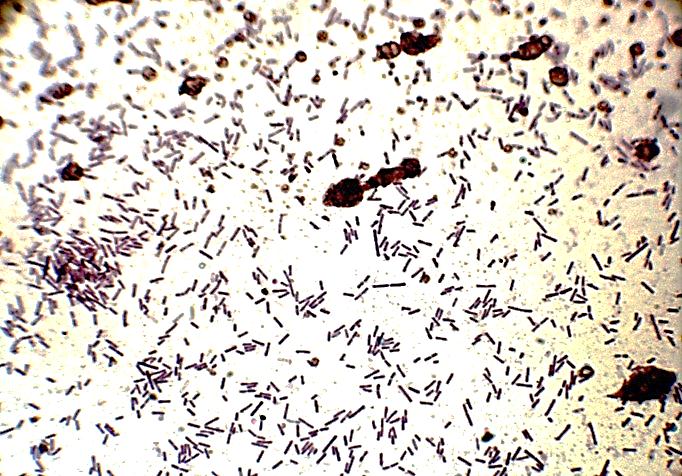

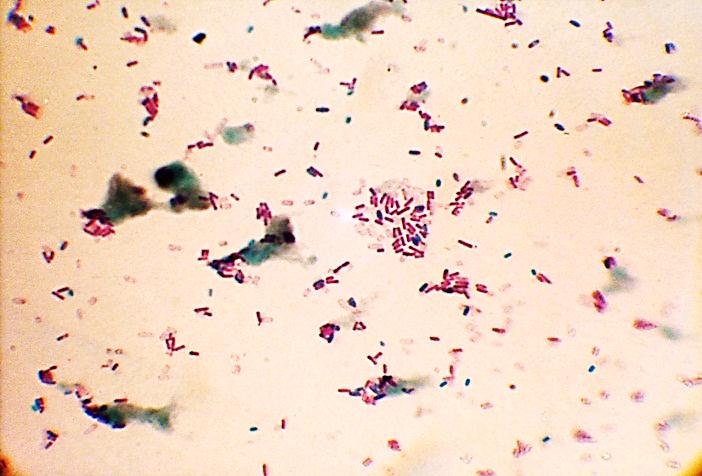

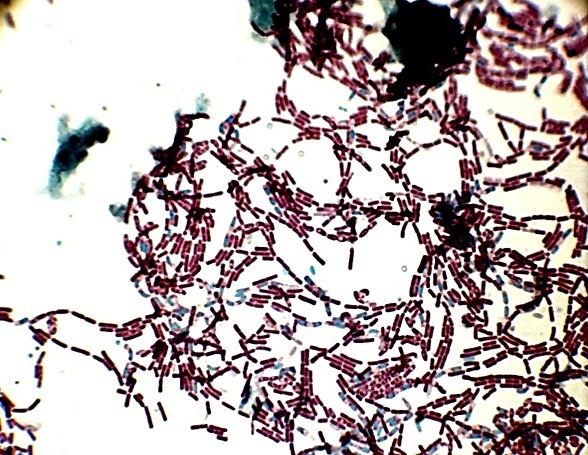

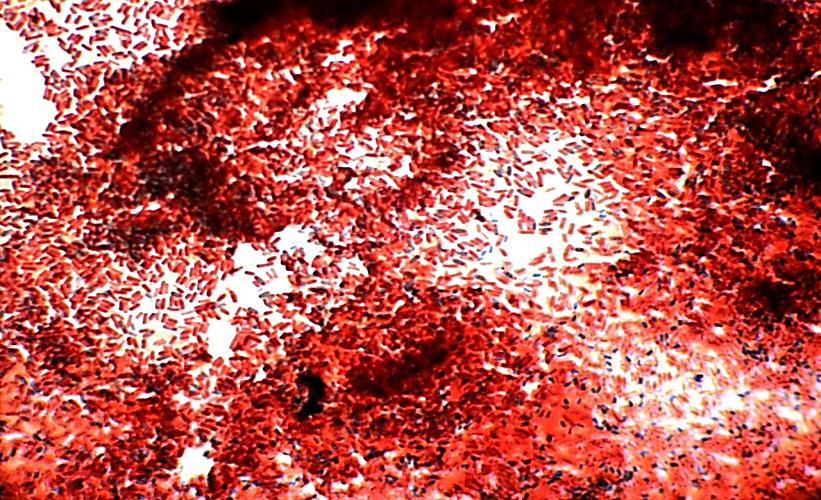

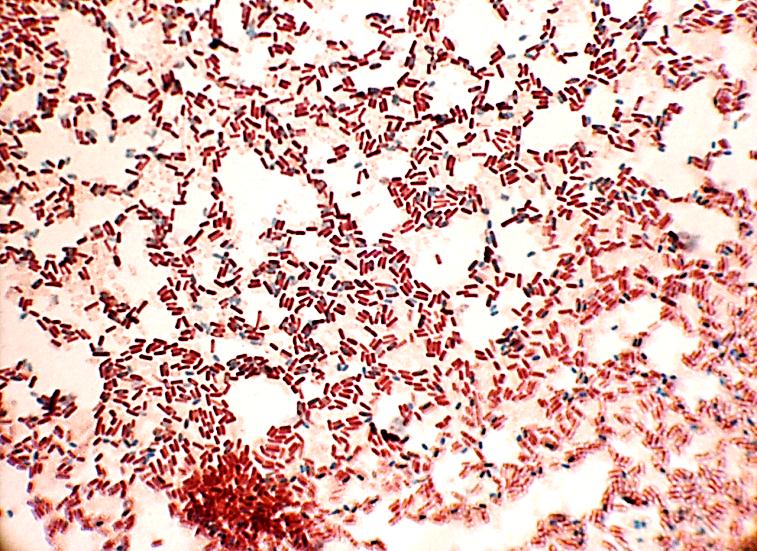

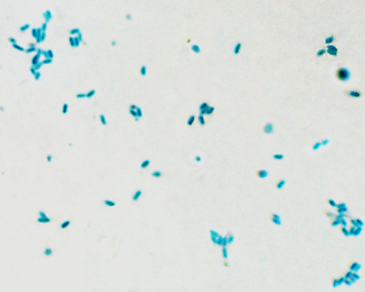


| **Preference parameters** | **Formaldehyde 1%** | **H_2_O_2_**  **1%** | **Acetic acid 1%** | **Pasteurization**  **70 ^o^C** | **Dry heat 120 ^o^C** | **Moist heat 120 ^o^C** | **UV irradiation 254 nm** | **Microwave 2.45 GHz** |
| --- | --- | --- | --- | --- | --- | --- | --- | --- |
| **Min/Max** | max | max | max | max | max | max | max | max |
| **Weight** | 1.00 | 1.00 | 1.00 | 1.00 | 1.00 | 1.00 | 1.00 | 1.00 |
| **Preference Fn.** | Linear | Linear | Linear | Linear | Linear | Linear | Linear | Linear |
| **Preference** | 100.00 | 100.00 | 100.00 | 100.00 | 100.00 | 100.00 | 100.00 | 100.00 |
| **Minimum** | 16.63 | 4.13 | 3.13 | 62.50 | 11.63 | 50.00 | 37.50 | 0.00 |
| **Maximum** | 56.33 | 88.00 | 96.00 | 100.00 | 77.50 | 77.50 | 94.55 | 16.59 |
| **Average** | 29.89 | 66.23 | 71.99 | 81.43 | 38.50 | 59.92 | 63.74 | 3.97 |
| **Standard Dev.** | 13.89 | 31.35 | 34.67 | 14.02 | 29.68 | 9.53 | 18.88 | 6.36 |
| ***C. perfringens*** | 24.00 | 80.00 | 96.00 | 100.00 | 16.00 | 56.00 | 55.00 | 0.00 |
| ***C. difficile*** | 22.50 | 75.00 | 87.50 | 95.00 | 77.50 | 77.50 | 71.25 | 2.00 |
| ***B. cereus*** | 30.00 | 84.00 | 90.00 | 76.92 | 15.39 | 61.54 | 60.38 | 0.00 |
| ***B. subtilis*** | 56.33 | 88.00 | 83.33 | 72.73 | 72.00 | 54.55 | 94.55 | 16.59 |
| ***B. amyloliquefaciens*** | 16.63 | 4.13 | 3.13 | 62.50 | 11.63 | 50.00 | 37.50 | 1.25 |

**Table S1. Preference parameters and the survival values of *Bacillus* and *Clostridium* spores after 10 mins treatment with selected sporicidal agent**

**Optimization of critical variables for sporicidal activity using Response Surface Methodology (RSM)**

## METHODOLOGY

## Experimental design of RSM for sporicidal activity

In order to achieve the maximum throughput of any process, optimization of process factors is of supreme focus. RSM explores the individual as well as interactional behavior of factors in a statistical approach towards maximized production by troubleshooting weak points^1^. Central composite design (CCD) matrix of RSM was employed to evaluate the influence of temperature, pH, treatment time and nanosilver concentration at five levels in the sporicidal experimental design. Five coded levels (–α, -1, 0, +1, +α) for each variable are shown in Table S2. A total of 30 trials including 16 factorial points, 8 axial points and 6 central points were carried out (Table S3).

**Table S2. Variables and their levels for the central composite experimental design**

| **Code** | **Variables** | **Range and levels** | | | | |
| --- | --- | --- | --- | --- | --- | --- |
|  |  | **-α** | **-1** | **0** | **+1** | **+α** |
| A | Temperature (^o^C) | 30 | 35 | 40 | 45 | 50 |
| B | pH | 5 | 6 | 7 | 8 | 9 |
| C | Treatment time (min) | 2 | 4 | 6 | 8 | 10 |
| D | Nanosilver (µg mL^-1^) | 0 | 25 | 50 | 75 | 100 |

**Experiment**

To estimate the sporicidal potential of nanosilver, around 10^5^ spores were coated on to a 1 cm^2^ sterile carrier glasses, dried and added to the 30 experimental sets. After the desire contact time, all the spore carriers were transferred to a sterile separate growth medium and incubated^2,3^ followed by which, the percentage of spore inhibition for each trial was calculated and the values were the response value (Y).

Table S3. Experimental factor levels and the respective sporicidal activity in percentage

| **Run** | **Type** | **Temp (˚C)** | **pH** | **Time (min)** | **Nanosilver (µg mL^-1^)** | ***B. cereus*** | ***B. amyloliquefaciens*** | ***B. subtilis*** | ***C. perfringens*** | ***C. difficile*** |
| --- | --- | --- | --- | --- | --- | --- | --- | --- | --- | --- |
|  | **Factors** | | | | | **% of inhibition (Y)** | | | | |
| 1  2  3  4  5  6  7  8  9  10  11  12  13  14  15  16  17  18  19  20  21  22  23  24  25  26  27  28  29  30 | Axial  Fact  Center  Fact  Center  Fact  Axial  Center  Fact  Fact  Fact  Axial  Fact  Axial  Fact  Fact  Center  Axial  Fact  Fact  Axial  Fact  Fact  Center  Axial  Fact  Fact  Fact  Center  Axial | 40  45  40  35  40  45  40  40  45  35  45  40  35  40  35  35  40  40  45  35  40  45  35  40  50  45  45  35  40  30 | 7  6  7  8  7  8  7  7  6  6  6  7  8  9  6  6  7  5  8  8  7  8  6  7  7  6  8  8  7  7 | 6  4  6  4  6  8  6  6  8  8  8  10  8  6  4  8  6  6  8  8  2  4  4  6  6  4  4  4  6  6 | 100  75  50  75  50  25  0  50  75  25  25  50  25  50  25  75  50  50  75  75  50  25  75  50  50  25  75  25  50  50 | 42.45  12  33.7  27  40.6  15  0.15  33.95  20.14  12.6  12.35  33.95  17.75  36  4.9  43.25  34  22.85  28.5  43.4  15  14.65  25.45  33  29.75  10.55  35.3  6.95  33.7  32.9 | 55.576  31.333  42.01  53.636  42.848  14.242  0.803  43.455  45.455  17.212  18.242  36  16.818  38.733  7.152  43.333  43.339  30.182  49.515  36.212  18.152  10.152  27.273  44.242  40.667  9.303  33.333  9.455  43.724  39.818 | 51.975  33.892  43.517  43.762  37.392  12.075  0.758  40.658  36.925  11.783  16.421  36.167  9.917  42.992  9.8  47.833  40.833  34.708  49.117  42.7  14.292  7.875  27.417  41.708  46.783  12.017  39.55  10.092  40.075  22.511 | 22.71  79.561  55.11  42.771  43.679  58.895  2.65  41.787  81.681  60.787  56.775  56.927  60.484  32.4  64.875  77.29  47.994  84.33  77.063  54.05  61.166  53.899  71.537  50.719  74.186  36.412  39.667  46.253  47.313  74.716 | 25.698  40.756  30.291  19.244  26.047  19.36  2.326  27.326  33.14  35.058  35.581  31.221  12.849  18.837  36.047  47.733  29.36  54.012  18.14  25.233  25.349  18.198  41.163  29.07  33.547  31.744  10.349  13.837  28.081  60.058 |

**Data analysis**

Design-Expert, Trial version 7.1.5 (Stat-Ease, Minneapolis, Minn, USA) was used for the regression analysis and for plotting the response surface. The significance and adequacy of polynomial model was assessed by coefficient of determination (R^2^) and analysis of variance (ANOVA). The statistical significance was predicted by correlation coefficient (R), Fisher’s statistical test (F-test) and t-test^1^.

**RESULTS AND DISCUSSION**

**Optimization of sporicidal conditions using RSM**

The influence of individual as well as combined effect of temperature, pH, contact time and nanosilver concentration on the bacterial endospores and the interaction among the factors were analyzed under central composite design matrix. The results acquired from the experimental runs (Table S3) were fed to the Design-Expert software to identify a suitable model. The experimental values of *B. cereus, B. amyloliquefaciens, B. subtilis, C. perfringens* and *C. difficile* spores were in good agreement with the response based predicted values. The coefficient of determination (R^2^) for *B. cereus, B. amyloliquefaciens, B. subtilis, C. perfringens* and *C. difficile* were 0.91, 0.93, 0.92, 0.85 and 0.91 respectively, which was quantitatively assessed by the correlation between the experimental value and the predicted responses. In general, the R^2^ value of a better model with high accuracy is closer to 1^1^. The R^2^ values indicated, 91%, 93%, 92%, 85% and 91% variations for *B. cereus, B. amyloliquefaciens, B. subtilis, C. perfringens* and *C. difficile* sporicidal activity by individual variables. Fit quality of a model is also measured by adjusted R^2^ value, which should be smaller than the R^2^ values^1^. The adjusted R^2^ values for *B. cereus, B. amyloliquefaciens, B. subtilis, C. perfringens* and *C. difficile* were, 0.83, 0.86, 0.84, 0.72 and 0.82, respectively. The significance of a model predicted from the F value is the ratio between the model mean square and the residual error^1^. The F values obtained for *B. cereus, B. amyloliquefaciens, B. subtilis, C. perfringens* and *C. difficile* were 11.13, 14.13, 12.22, 6.21, and 10.60, respectively, which were greater than the tabulated F value (2.403 at 95% significance) substantiating the competence of the model fitness.

The response surface 2D contour plots (Fig. S2) were generated from varying the range of two factors within the design level by keeping the other two at their zero range. The optimal sporicidal conditions and the predicted sporicidal values were determined from the response surface plots (Table S4) with the desirability value close to 1. These sporicidal values were verified experimentally.

**Figure S2. Response surface 2D contour plots of sporicidal activity against *Bacillus* and *Clostridium* endospores.** Response plots of spores (a) *B. cereus,* (b) *B. amyloliquefaciens,* (c) *B. subtilis,* (d) *C. perfringens* and (e) *C. difficile* and the interaction between process parameters such as pH with temp (a_1_, b_1_, c_1_, d_1_ and e_1_), time with temp (a_2_, b_2_, c_2_, d_2_ and e_2_), nanosilver with temp (a_3_, b_3_, c_3_, d_3_ and e_3_), time with pH (a_4_, b_4_, c_4_, d_4_ and e_4_), nanosilver with pH (a_5_, b_5_, c_5_, d_5_ and e_5_) and nanosilver with temp (a_6_, b_6_, c_6_, d_6_ and e_6_), is displayed below.


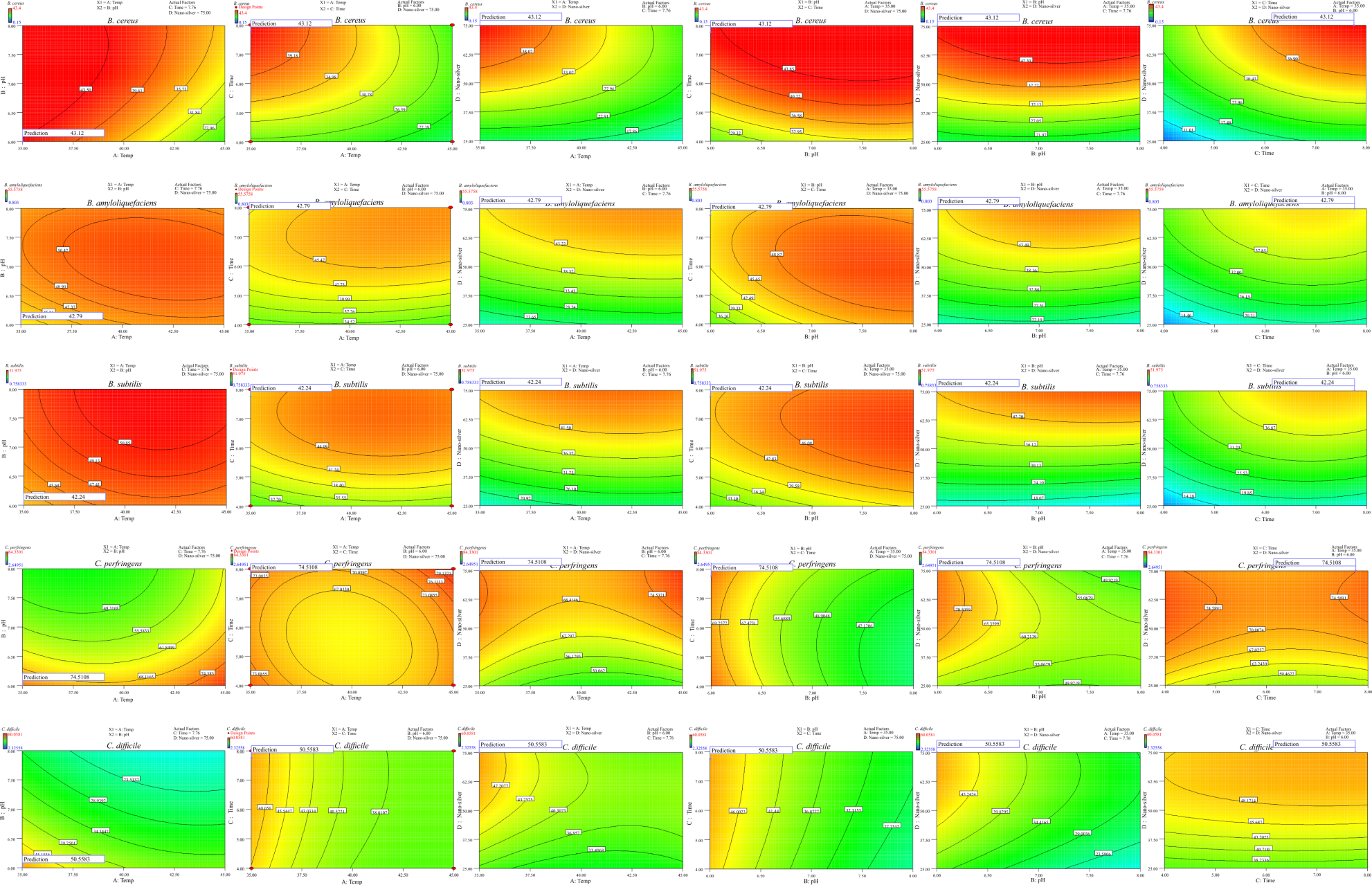


a_1_

a_2_

a_3_

a_4_

a_5_

a_6_

b_1_

b_2_

b_3_

b_4_

b_5_

b_6_

c_1_

c_2_

c_3_

c_4_

c_5_

c_6_

d_1_

d_2_

d_3_

d_4_

d_5_

d_6_

e_1_

e_2_

e_3_

e_4_

e_5_

e_6_

Table S4. RSM- optimized condition and the predicted sporicidal values.

| **Temp**  **(˚C)** | **pH** | **Time**  **(min)** | **Nanosilver**  **(µg mL^-1^)** | ***B. cereus*** | ***B. amyloliquefaciens*** | ***B. subtilis*** | ***C. perfringens*** | ***C. difficile*** | **Desirability** |
| --- | --- | --- | --- | --- | --- | --- | --- | --- | --- |
| 35.00 | 6.00 | 7.78 | 75.00 | 43.14 | 42.75 | 42.22 | 74.58 | 50.56 | 0.854 |

Table S5. Preference parameters and sporicidal percentage of selected sporicides after 10 mins treatment

| **Preference parameters** | ***C. perfringens*** | ***C. difficile*** | ***B. cereus*** | ***B. subtilis*** | ***B. amyloliquefaciens*** |
| --- | --- | --- | --- | --- | --- |
| **Min/Max** | max | max | max | max | max |
| **Weight** | 1.00 | 1.00 | 1.00 | 1.00 | 1.00 |
| **Preference Fn.** | Linear | Linear | Linear | Linear | Linear |
| **Preference** | 100.00 | 100.00 | 100.00 | 100.00 | 100.00 |
| **Minimum** | 0.00 | 5.00 | 10.00 | 5.45 | 37.50 |
| **Maximum** | 100.00 | 98.00 | 100.00 | 83.41 | 98.75 |
| **Average** | 51.00 | 39.36 | 50.11 | 34.15 | 77.25 |
| **Standard Dev.** | 35.27 | 30.22 | 30.16 | 22.30 | 20.86 |
| **Formaldehyde 1%** | 76.00 | 77.50 | 70.00 | 43.67 | 83.37 |
| **H_2_O_2_ 1%** | 20.00 | 25.00 | 16.00 | 12.00 | 95.87 |
| **Acetic acid 1%** | 4.00 | 12.50 | 10.00 | 16.67 | 96.87 |
| **Pasteurization 70 ^o^C** | 0.00 | 5.00 | 23.08 | 27.27 | 37.50 |
| **Dry heat 120 ^o^C** | 84.00 | 22.50 | 84.61 | 28.00 | 88.37 |
| **Moist heat 120 ^o^C** | 44.00 | 22.50 | 38.46 | 45.45 | 50.00 |
| **UV radiation 254 nm** | 45.00 | 28.75 | 39.62 | 5.45 | 62.50 |
| **Microwave 2.45 GHz** | 100.00 | 98.00 | 100.00 | 83.41 | 98.75 |
| **Nanosilver** | 86.00 | 62.50 | 69.23 | 45.45 | 82.00 |


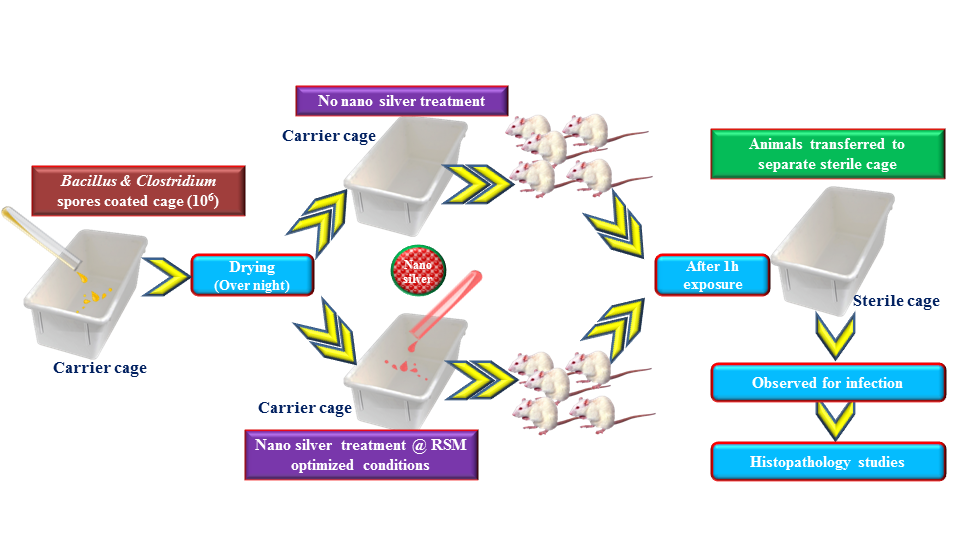
**Figure S3. Schematic representation of environmental spore disinfection using nanosilver**

**Figure S4. Histopathology of lung tissues in mice infected with spores of *B. cereus.*** Black arrows identify the interstitial encapsulated *B. cereus.* Sections were stained with hematoxylin-

eosin.

**
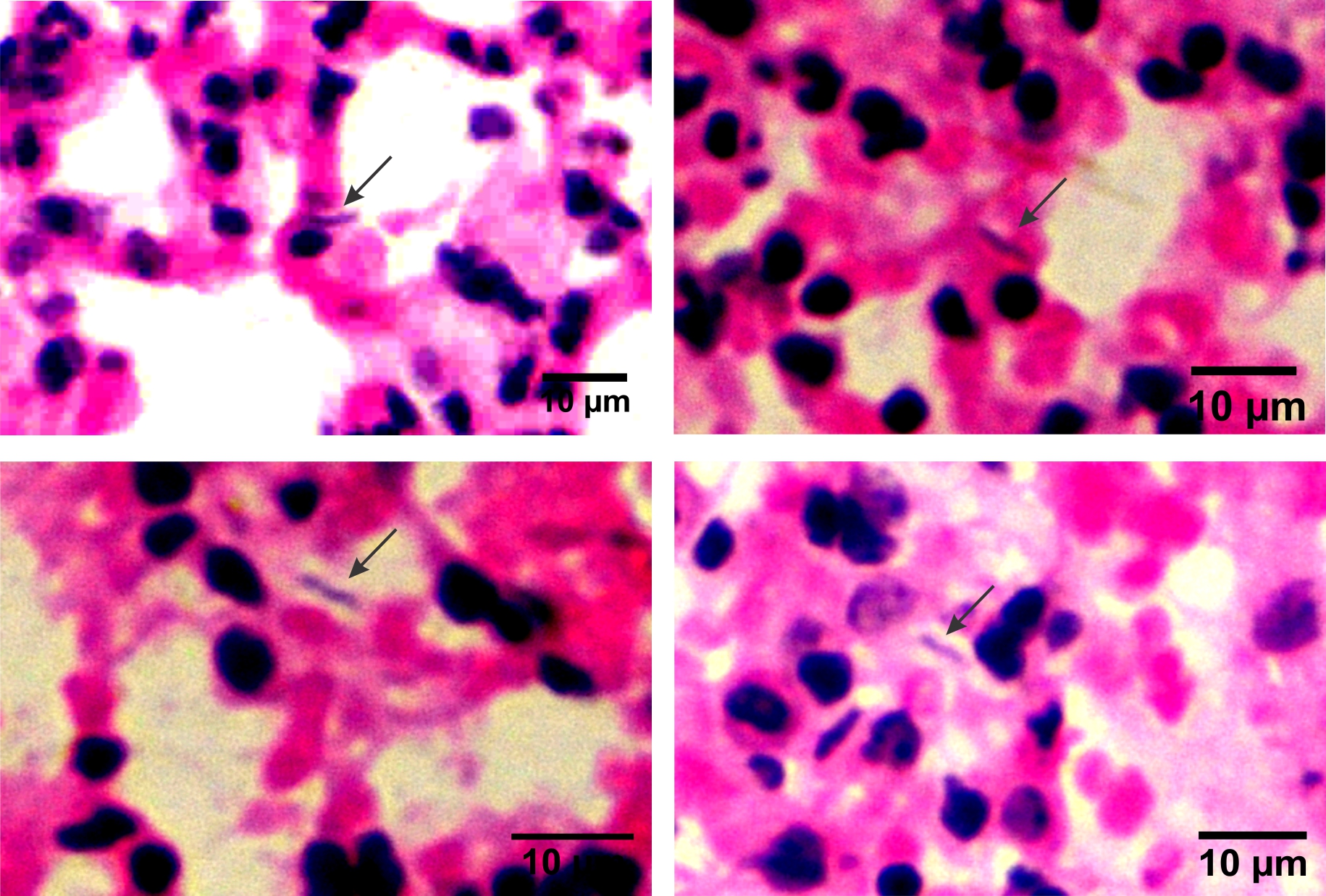
**

**References**

1 Kasiri, M. B., Modirshahla, N. & Mansouri, H. Decolorization of organic dye solution by ozonation; Optimization with response surface methodology. *International Journal of Industrial Chemistry* **4**, 1-10 (2013).

2 Humphreys, P. N. Testing standards for sporicides. *J. Hosp. Infect.* **77**, 193-198 (2011).

3 Tomasino, S. F. *et al.* Use of Alternative Carrier Materials in AOAC Official Method SM 2008.05, Efficacy of Liquid Sporicides Against Spores of Bacillus subtilis on a Hard, Nonporous Surface, Quantitative Three-Step Method. *J. AOAC Int.* **93**, 259-276 (2010).
